# *Gochujang* elicits anti-cancer effects via distinct molecular mechanisms across different gastrointestinal cancer cell types

**DOI:** 10.29219/fnr.v70.13691

**Published:** 2026-04-22

**Authors:** Chan-Ho Park, Eun-Bi Seo, Su-Bin Lee, Anna Han

**Affiliations:** 1 Department of Food Science and Human Nutrition, Jeonbuk National University, Jeonju, Jeollabukdo, Republic of Korea; 2 K-Food Research Center, Jeonbuk National University, Jeonju, Jeollabukdo, Republic of Korea

**Keywords:** Gochujang, anti-cancer, gastrointestinal cancer cell type, cell survival, ROS metabolism

## Abstract

Gastrointestinal (GI) cancers, including gastric, hepatic, and pancreatic cancers, account for a substantial proportion of cancer-related global morbidity and mortality. Dietary habits and bioactive food components significantly influence cancer initiation and progression. As a representative Korean traditional fermented food (KTFF), *Gochujang* exhibits multiple health benefits, including anti-mutagenic effects; however, its detailed cellular and molecular mechanisms of anti-cancer effects across different GI cancer cell types remain unclear.

The radical scavenging activity of *Gochujang* extract (GE) was ABTS: 2.86–31.84% and DPPH: 7.95–52.79%. In addition, total phenolic and flavonoid contents of GE were 6.94 ± 0.23 mg GAE/g and 9.28 ± 0.72 mg RE/g, respectively. GE markedly inhibited cell viability, migration, and colony formation in all GI cancer cell lines, including gastric cancer (AGS and SNU-668), hepatic cancer (HepG2 and Hep3B), and pancreatic cancer (MIA PaCa-2). Furthermore, GE markedly reduced the expression of cell proliferation-related proteins and changed the levels of apoptosis-involved proteins in AGS, Hep3B, and MIA PaCa-2 cells, indicating its multifaceted anti-cancer activity. Interestingly, GE significantly altered the expression of antioxidant enzymes and increased reactive oxygen species (ROS) production in gastric and hepatic cancer cells, but these effects of GE were not observed in pancreatic cancer cells. In conclusion, these findings demonstrate that *Gochujang* exerts comprehensive anti-cancer effects across gastric, hepatic, and pancreatic cancer cells through the coordinated inhibition of proliferation-, migration-, and survival-related mechanisms, while additionally regulating ROS metabolism in a distinctly cell type–specific manner.

## Popular scientific summary

- *Gochujang* significantly inhibits cell viability, migration, and colony formation in multiple gastrointestinal (GI) cancer cell types.
- *Gochujang* modulates key regulators of cell proliferation and apoptosis, indicating its involvement in core anti-cancer mechanisms.
- Notably, *Gochujang* differentially regulates antioxidant enzyme expression and reactive oxygen species (ROS) production in a cell type–dependent manner.

Digestive system-related cancers, including gastric, hepatic, and pancreatic cancers, account for a high proportion of cancer-related morbidity and mortality, collectively contributing to about 20% of global cancer deaths (1, 2). While genetic predisposition of individuals plays a critical role in cancer development, dietary habits are also significantly responsible for increased cancer risk and aggressive cancer progression (3). For example, numerous epidemiological studies have consistently demonstrated that high consumption of processed and red meats, excessive fat intake, and low consumption of fruits and vegetables are strongly associated with an increased risk of gastrointestinal (GI) cancers (4, 5). Conversely, a diet containing diverse antioxidants and bioactive compounds (e.g. a plant-based diet) is inversely correlated with cancer development and progression (6). Therefore, understanding the role of diet and/or diverse functional compounds of foods in cancer development is necessary to establish effective preventive strategies and reduce further aggressive cancer progression.

Previous studies have demonstrated that bioactive compounds can have anti-cancer effects by modulating various molecular and cellular mechanisms, depending on the types of cancer cells (7–10). For example, curcumin suppresses proliferation and induces apoptosis in gastric carcinoma AGS cells, while it causes mitochondrial dysfunction and activates endoplasmic reticulum (ER) stress pathways to exert its anti-cancer effects in colorectal carcinoma HT-29 cells (7). Berberine exerts anti-cancer effects by inducing of G2/M phase cell cycle arrest, cellular aging, and autophagy in glioblastoma U343 cells; on the other hand, in pancreatic carcinoma MIA PaCa-2 cells, it causes G1 phase cell cycle arrest and a significant inhibition of cell migration and invasion (8). These findings suggest that the anti-cancer effects of certain bioactive compounds target distinct pathways and modulate different molecular mechanisms, depending on the specific cell type, rather than relying on a single common mechanism. Thus, a study examining the anti-cancer effects of specific foods and/or functional compounds in various cancer cell types is necessary to accurately understand their effects and to develop more effective cancer type-specific dietary strategies.

As a global representative of symbiotic food, Korean traditional fermented foods (KTFF) contain beneficial microbes and functional compounds, providing various health beneficial effects (11–17). For example, *Gochujang* improves intestinal health by maintaining gut microbiota balance in the rat colitis model (17). Additionally, *Gochujang* has been shown to improve obesity by activating brown adipose tissue metabolism (11, 18). Previously, the potential of *Gochujang*’s anti-cancer effects has been simply screened in different cancer cell lines, including gastric, colorectal, and lung cancer cells, by measuring its effects on cell viability (19); however, the exact molecular and cellular mechanisms to understand *Gochujang*’s anti-cancer effects, depending on different GI cancer cell types, have not been fully established yet. A recent study reports that *Gochujang* exerts anti-cancer effects in colorectal cancer cell lines by causing an imbalance in reactive oxygen species (ROS) metabolism through the downregulation of antioxidant enzyme expression, including heme oxygenase-1 (HO-1), superoxide dismutase-1 (SOD-1), and superoxide dismutase-2 (SOD-2) (20). However, comparative studies on whether *Gochujang* inhibits distinct molecular mechanisms in different GI cancer cell types to exert its anti-cancer effects are unexplored.

Therefore, this study aimed 1) to investigate the anti-cancer effects of *Gochujang* across different GI cancer cell types by integrating its impacts on cell viability, migration, and colony formation, 2) to compare the cellular and molecular mechanisms for its anti-cancer effects in different GI cancer cell types, thereby clarifying the cancer cell type-specific anti-cancer mechanisms of *Gochujang*.

## Materials and methods

### Preparation of GE

*Gochujang* samples collected from Ganghwa-gun, Sunchang-gun, and Yeongwol-gun in South Korea were blended and then extracted. The combined sample was freeze-dried, pulverized, and extracted with 1 L of 80% ethanol at room temperature under constant stirring. After standing, the first supernatant was carefully collected without centrifugation. The remaining pellet was re-extracted by adding 80% ethanol at a tenfold volume and stirring again. This secondary extract was combined with the first. The mixture was filtered using a vacuum filtration system, concentrated via rotary evaporation, and subsequently freeze-dried for approximately 4 days to yield a dry powder. The powder was stored at –70°C until further use. For cellular experiments, the powder was dissolved in dimethyl sulfoxide (DMSO), which had been diluted to 1% in phosphate-buffered saline (PBS). The solution was passed through a 0.45 μM syringe filter before application to cells.

### Cell culture

AGS, SNU-668, and MIA PaCa-2 cells were cultured in RPMI-1640 MEDIUM (HyClone, U.S.A.), HepG2, and Hep3B cells were cultured in DMEM MEDIUM (HyClone, U.S.A.). The culture medium was supplemented with 100 IU/mL penicillin, 100 μg/mL streptomycin (SIGMA, USA), and 10% heat-inactivated fetal bovine serum (FBS) (Gibco, U.S.A.), which had been treated in a water bath. All cell lines were cultured at 37°C in a humidified incubator maintained with 5% CO_2_ and 95% air.

### Radical scavenging activity of GE

The antioxidant activity of GE was determined using 2,2′-azino bis (3-ethylbenzothiazoline-6-sulfonic acid) (ABTS) and 2,2-diphenyl-1-picrylhydrazyl (DPPH) radical scavenging assays. For the ABTS assay, 7.4 mM ABTS and 2.45 mM potassium persulfate were mixed and kept in the dark at room temperature overnight. The mixture was then diluted to an absorbance of 0.7–0.8 at 734 nm. A total of 180 μL of the ABTS solution was added to 20 μL of GE sample or Trolox standard in a 96-well plate, followed by incubation in the dark for 10 min. Absorbance was measured at 734 nm. For the DPPH assay, a 0.2 mM DPPH solution in ethanol was prepared and kept in the dark. Then, 160 μL of the DPPH solution and 40 μL of GE sample or Trolox were mixed in a 96-well plate and incubated for 35 min in the dark. Absorbance was measured at 517 nm using a microplate reader (TECAN, INFINITE M PLEX) with i-control 2.0 software.

### Assessment of total polyphenol and total flavonoid contents

Gallic acid was used as the standard for total phenolic content, and rutin was used as the standard for total flavonoid content. The absorbance for the TPC was 760 nm and the TFC was 420 nm, respectively. The Absorbance was read by i-control 2.0 software of the microplate reader (TECAN, INFINITE M PLEX).

### Cell viability

To evaluate the cytotoxic effects of GE on various cancer cell lines, including AGS, SNU-668, HepG2, Hep3B, and MIA PaCa-2, cell viability was assessed using the EZ-CYTOX assay kit (DoGenBio, Seoul, Korea). Cells were seeded in 96-well plates (SPL, USA) at appropriate densities (5 × 10^3^ cells/well), and incubated for 24 h to allow for attachment. Following incubation, the culture medium was replaced with fresh medium containing GE at varying concentrations (0, 0.25, 0.5, 1, 5, 7.5, and 10 mg/mL), and cells were treated for 24, 48 h. After determining the IC_50_ values specific to each cell line, GE was further applied at concentrations tailored to each line and the respective experimental conditions. Post-treatment, the medium was discarded and replaced with a 1:10 mixture of EZ-CYTOX reagent and culture medium. A total of 100 μL of this mixture was added to each well, and the plate was incubated for 1 h. After gentle shaking, absorbance was measured at 450 nm using the i-control 2.0 software with the INFINITE M PLEX microplate reader (TECAN). Cell viability was expressed as a percentage relative to the untreated control group.

### Wound healing assay

Cells were plated in 6-well plates (SPL, Pocheon-si, Korea) and allowed to adhere for 24 h. Once confluence was reached, a linear wound was created at the center of each well using sterile pipette tips. The culture medium was then carefully removed and replaced with fresh medium containing GE. Cells were treated for a total of 72 h, with the treatment medium renewed daily. At 0 and 72 h post-treatment, wound closure was documented by capturing images using the EVOS™ M7000 Imaging System (ThermoFisher, AMF7000). The wound healing process was quantified by measuring the gap distance between the migrating cell fronts using ImageJ software.

### Colony formation assay

A total of 2,000 cells were seeded into each well of a 6-well plate and incubated overnight at 37°C to allow for cell attachment. The following day, cells were treated with varying concentrations of GE: 0, 0.5 and 2.5 for AGS, SNU-668, Hep3B and MIA PaCa-2, and 0, 2.5 and 10 mg/mL for HepG2 cells. The culture medium containing GE was replenished every two days. After 10 days of continuous culture, the wells were gently rinsed twice with PBS to remove non-adherent cells. Colonies were then fixed and stained using a 0.2% crystal violet solution for 2 h at room temperature. Subsequently, excess stain was removed by washing each well three times with distilled water. Colony formation was visualized, and colony numbers were quantified using ImageJ software.

### Western blot

Cell protein samples were extracted using RIPA buffer. The samples were subjected to centrifugation at 15,000 g for 15 min, performed twice, to separate the supernatant. Subsequently, the concentration of all samples was adjusted accordingly. Proteins were separated using an 8–15% SDS-PAGE gel. Proteins were separated using an 8–15% SDS-PAGE gel, followed by overnight incubation with the primary antibody. The following primary antibodies were used: *β*-Actin (#A2066) from Sigma-Aldrich Inc. (Gangnam-gu, Seoul); heme oxygenase 1 (HO-1) (#sc-390991) from SANTA CRUZ Biotechnology, Inc. (Dallas, TX, USA); phosphorylated signal transducer and activator of transcription 3 (p-STAT3) (#4113), p-Rb (#8516), Cyclin B1 (#4138), cyclin-dependent kinase 2 (CDK2) (#2546), B-cell lymphoma 2 (Bcl-2) (#3498), B-cell lymphoma-extra large (Bcl-xL) (#2764), Bcl-2 Interacting Mediator of cell death (Bim) (#2933), superoxide dismutase 1 (SOD1) (#37385), superoxide dismutase 2 (SOD2) (#13141), and Catalase (#14097) from Cell Signaling Technology, Inc. (Danvers, Massachusetts, USA). The signal was detected by ECL solution, and expression was supported by a CehmiDoc KwikQuant imaging system (Kindle Bioscences LLC, Boston, MA).

### ROS assay

ROS levels were assessed using a 2,7-dichlorodihydrofluorescein diacetate (DCFDA) cellular ROS assay kit (ab113851; Abcam, USA) following the manufacturer’s instructions. AGS, SNU-668, HepG2, Hep3B, and MIA PaCa-2 cells were seeded into 96 well plates at a density of 5 × 10^3^ cells/well. After allowing the cells to adhere for 24 h, GE was treated at 0 and 0.5 mg/mL for 24 h. 20 μM 2,7-DCFDA was then added and incubated in the dark for 45 min prior to the endpoint. A fluorescence intensity was detected at 485 nm excitation and 535 nm emission using the INFINITE M PLEX microplate reader (TECAN) with i-control 2.0 software. Fluorescent images were obtained with an EVOS^™^ M7000 Imaging System (ThermoFisher, AMF7000).

### Statistical analysis

All data from *in vitro* experiments were performed in triplicate and presented as the mean and standard error of the mean (SEM) of three independent experiments (*n* = 3). Statistical significance was calculated using Student’s *t*-test, and determined at *P < 0.05, ** *P* < 0.01, *** *P* < 0.001.

## Results

### GE has antioxidant activities

Before analyzing the effects of GE on GI cancer cell viability, its antioxidant capacities were primarily assessed. The radical scavenging activities of GE were determined by assessing ABTS and DPPH assays at concentrations ranging from 0.625 to 10 mg/mL. The ABTS scavenging activity of GE was 2.86–31.84%, DPPH scavenging activity was 7.95–52.79% (Supplementary Fig. 1A). Moreover, the total phenolic content of GE (10 mg/mL) was determined to be 6.94 ± 0.23 mgGAE/g, while the total flavonoid content was 9.28 ± 0.72 mgRE/g (Supplementary Fig. 1B).

### The effects of GE on cell viability in different GI cancer cell types

**Fig. 1:**
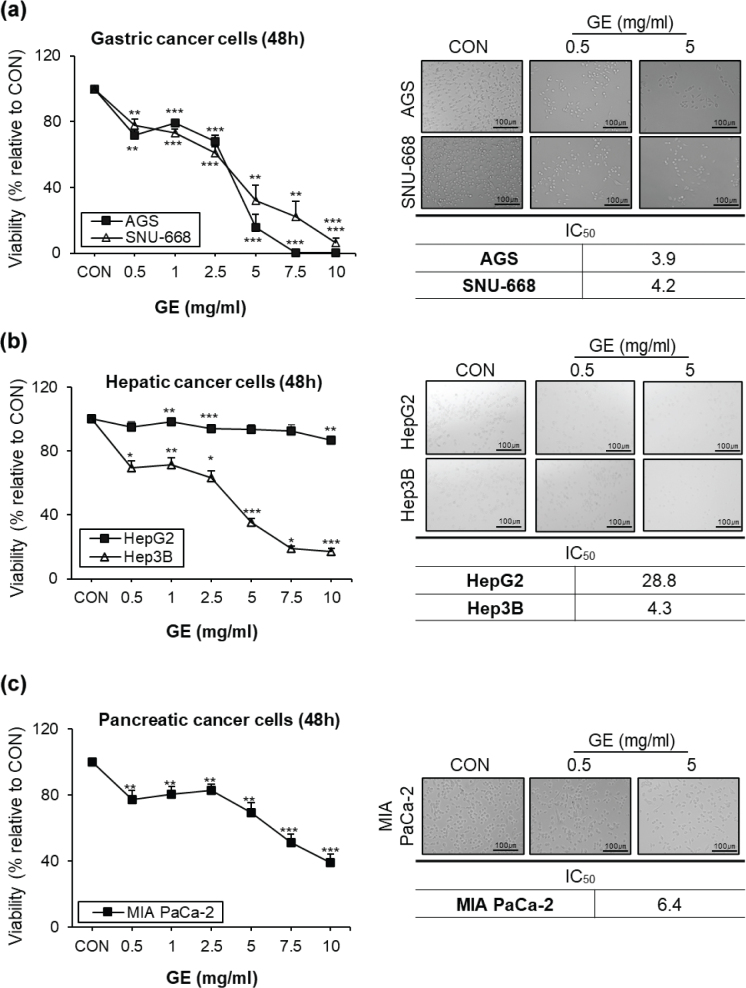
The effects of GE on cell viability across different GI cancer cell types. After 48 h of GE treatment following results show. (a) Cell viability, IC_50_, and representative image in AGS and SNU-668 cell lines. (b) Cell viability assay and IC_50_ in HepG2 and Hep3B cell lines. (c) Cell viability assay and IC_50_ in MIA PaCa-2 cell lines. The results were expressed as a percentage of viable cells compared to CON. Values are mean ± SEM (*n* = 3). scale bar = 100 μm. A *t*-test was performed to evaluate the statistical significance of differences. * (*P* < 0.05), ** (*P* < 0.01), *** (*P* < 0.001).

To test the anti-cancer effects of GE in different GI cancer cell lines, cell viability was measured. A significant reduction in cell viability was observed after GE treatment (48 h) across various GI cancer cell types (Fig. 1). GE significantly decreased the viability of gastric cancer cell lines, AGS and SNU-668. AGS exhibited greater sensitivity to GE than SNU-668: The IC_50_ values were 3.9 mg/mL for AGS and 4.2 mg/mL for SNU-668 (Fig. 1a). GE also markedly reduced the viability of hepatic cancer cell lines, HepG2 and Hep3B (Fig. 1b). Between two hepatic cancer cell lines, Hep3B cells were more sensitive to GE, with IC_50_ values of 28.8 mg/mL for HepG2 and 4.3 mg/mL for Hep3B. In addition, GE also suppressed the viability of the pancreatic cancer cell line MIA PaCa-2 (Fig. 1c), with an IC_50_ of 6.4 mg/mL. In addition, shorter GE treatment (24 h) also strongly reduced the viability of gastric cancer cell lines AGS and SNU-668, with IC_50_ values of 4.1 and 6.7 mg/mL, respectively (Supplementary Fig. 2A, left panel). Moreover, GE markedly decreased the viability of hepatic cancer cell lines HepG2 and Hep3B, with IC_50_ values of 17 and 5.6 mg/mL, respectively (Supplementary Fig. 2A, middle panel). In addition, GE significantly suppressed the viability of the pancreatic cancer cell line MIA PaCa-2, with IC_50_ value of 7.6 mg/mL (Supplementary Fig. 2A, right panel). Based on the cell line–dependent IC_50_ values obtained from the viability assay, selected treatment concentrations were used for subsequent experiments. For colony formation and Western blot analyses, doses around the IC_50_ were used to induce measurable cytotoxic and signaling changes without causing complete cell death, whereas a lower concentration was chosen for ROS levels to minimize non-specific oxidative stress associated with extensive cell loss. In conclusion, these observations demonstrate that GE significantly inhibits the cell viability of different types of GI cancer cells.

### The effects of GE on the ability of cell migration and colony formation across different GI cancer cell types

The migration assay assesses cancer cell motility, while the colony formation assay evaluates the ability of single cancer cells to proliferate and form colonies; thus, these indicators collectively provide an overall assessment of the metastatic potential of cancer cells (21).

**Fig. 2:**
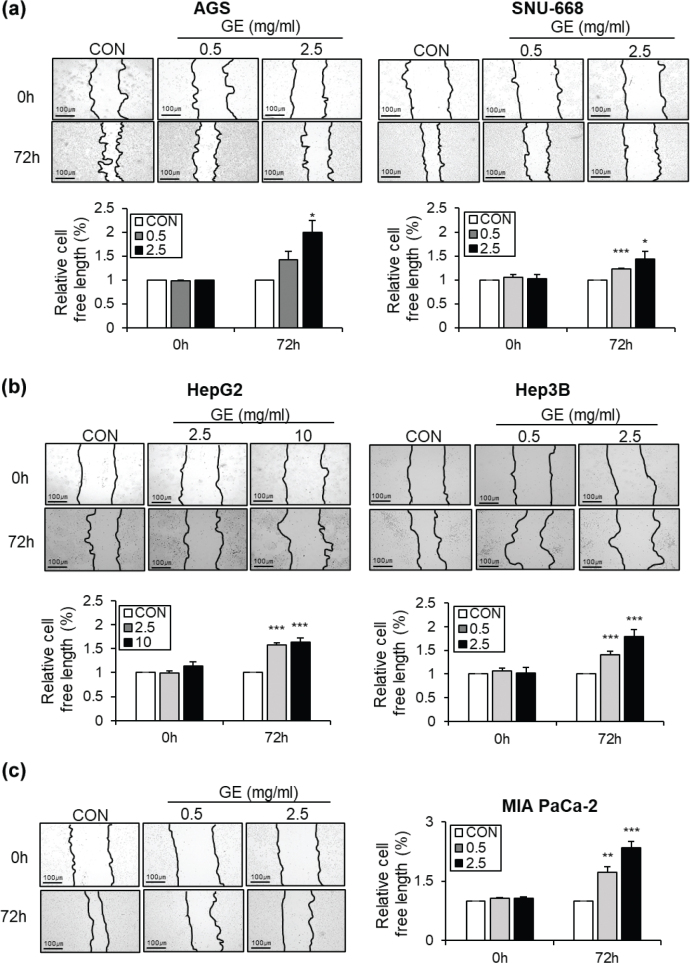
The effects of GE on cell migration across different GI cancer cell types. GE inhibited migration in diverse GI cancer cell lines. (a) Migration assay on AGS and SNU-668 cell lines. The cells received GE treatment at final concentrations of 0, 0.5, and 2.5 mg/mL. (b) Migration assay on HepG2 and Hep3B cell lines. The cells received GE treatment at final concentrations of 0, 2.5, and 10 mg/mL (HepG2) and 0, 0.5, and 2.5 mg/mL (Hep3B). (c) Migration assay on MIA PaCa-2 cell lines. The cells received GE treatment at final concentrations of 0, 0.5, and 2.5 mg/mL. Scratch area measured 0 and 72 h. Migration images of cells, and quantification bar graphs of relative cell-free length (%). Values are mean ± SEM (*n* = 3). scale bar = 100 μm. A *t*-test was performed to evaluate the statistical significance of differences. * (*P* < 0.05), ** (*P* < 0.01), *** (*P* < 0.001).

Since GE markedly reduced cancer cell viability (Fig. 1), a wound healing assay was conducted to evaluate the anti-cancer effects of GE on cell migration ability. GE markedly inhibited the migratory ability of gastric cancer cell lines (AGS and SNU-668) in a dose-dependent manner compared to CON (Fig. 2a). In both hepatic cancer cell lines (HepG2 and Hep3B), GE also significantly and dose-dependently reduced migration ability compared with CON (Fig. 2b). In addition, compared to CON, GE also strongly decreased the migration of pancreatic cancer cell line MIA PaCa-2 (Fig. 2c).

**Fig. 3:**
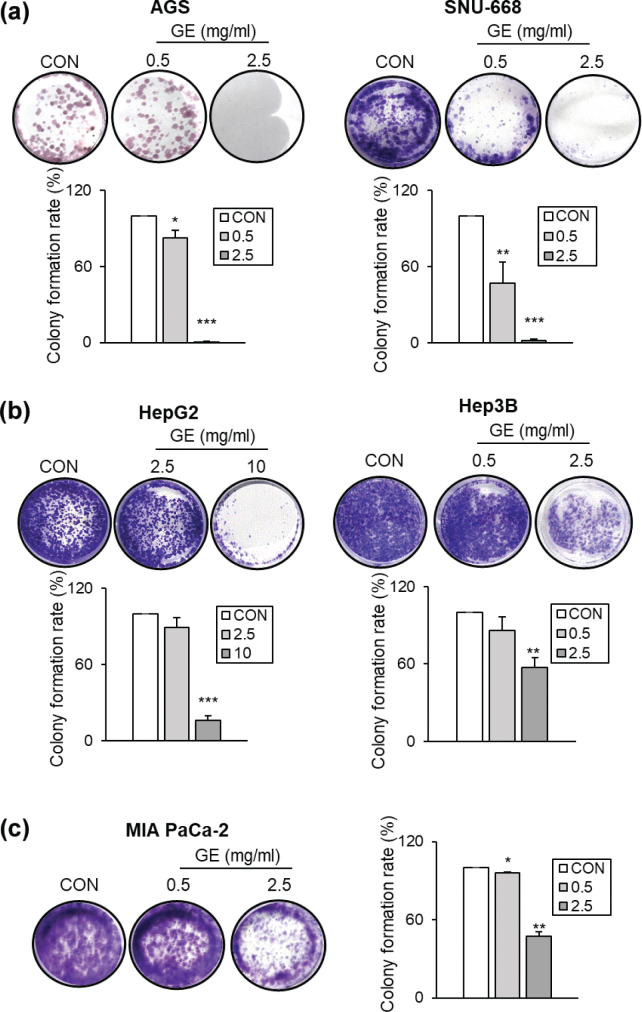
The effects of GE on colony formation across different GI cancer cell types. Colony formation images and bar graph (a) AGS and SNU-668, (b) HepG2 and Hep3B, and (c) MIA PaCa-2. Colony formation ability was expressed as a percentage of CON. Values are mean ± SEM (*n* = 3). A *t*-test was performed to evaluate the statistical significance of differences. * (*P* < 0.05), ** (*P* < 0.01), *** (*P* < 0.001).

Next, the effects of GE on colony formation were further investigated. In gastric cancer cell lines (AGS and SNU-668), GE led to a significant, dose-dependent reduction in colony formation compared to CON (Fig. 3a). In hepatic cancer cell lines (HepG2 and Hep3B), GE also markedly lowered colony formation in a dose-dependent manner compared with CON (Fig. 3b). Moreover, GE also suppressed the colony formation of the pancreatic cancer cell line MIA PaCa-2 compared with CON (Fig. 3c). In summary, GE strongly inhibits cancer cell migration and suppresses the colony-forming capability of various GI cancer cell types, suggesting its suppressive effects on the potential of cancer cells’ metastatic capabilities.

### The effects of GE on the protein levels involved in cell survival and cell death across different GI cancer cell types

The balance between cell cycle regulation and apoptosis is a fundamental biological system to maintain cell survival, which is primarily investigated to evaluate the anti-cancer effects of certain foods and/or bioactive compounds (22). Therefore, the effects of GE on protein expression involved in cell cycle regulation and apoptosis were probed.

**Fig. 4:**
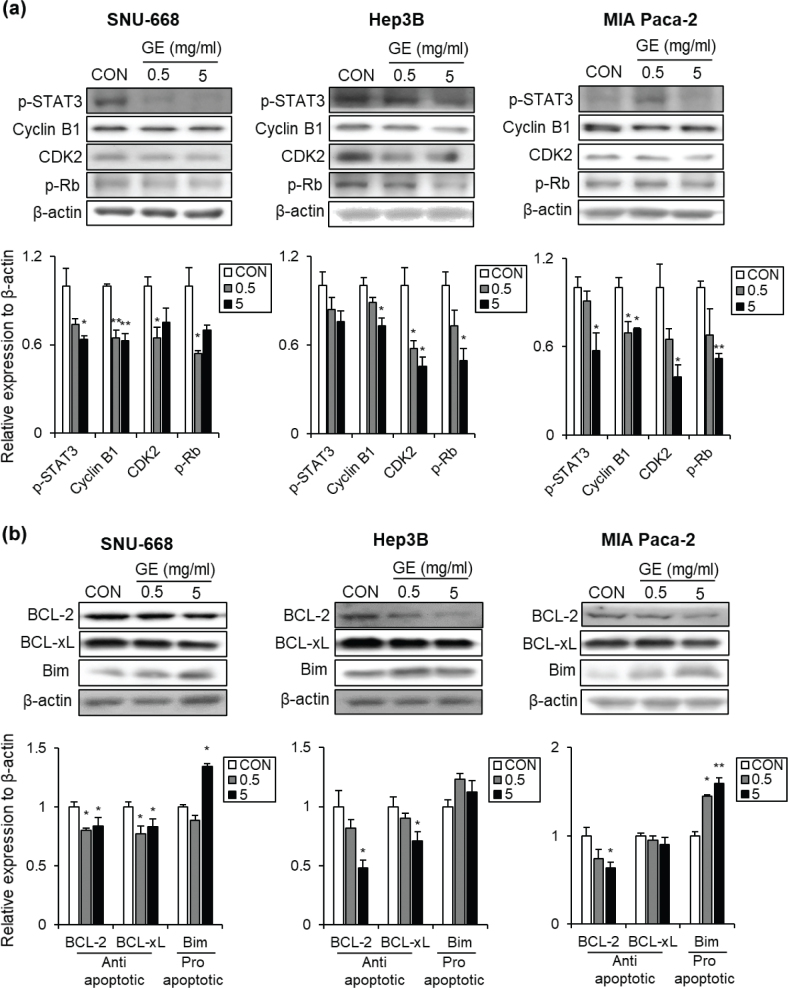
The effects of GE on the protein expression related to cell survival mechanisms across different GI cancer cell types. (a) Cell proliferation-related protein levels: Western blot images, the quantification bar graphs of SNU-668, Hep3B, and MIA PaCa-2. (b) Cell apoptosis-related protein levels: Western blot images, the quantification bar graphs of SNU-668, Hep3B, and MIA PaCa-2. *β*-Actin was used as a loading control. Values are mean ± SEM (*n* = 3). A *t*-test was performed to evaluate the statistical significance of differences. * (*P* < 0.05), ** (*P* < 0.01), *** (*P* < 0.001).

As shown in Fig. 4a, GE altered the expression of cell cycle-associated proteins in all types of GI cancer cells. In SNU-668 cells, GE significantly downregulated the protein levels of p-STAT3, cyclin B1, CDK2, and p-Rb in a dose-dependent manner (Fig. 4a, left panel). In Hep3B cells, GE lowered p-STAT3 expression without statistical significance, while it led to a significant decrease in cyclin B1, CDK2, and p-Rb levels at 5 mg/mL (Fig. 4a, middle panel). With MIA PaCa-2 cells, GE markedly suppressed the expression of p-STAT3, cyclin B1, CDK2, and p-Rb in a dose-dependent manner (Fig. 4a, right panel).

As presented in Fig. 4b, GE also significantly changed the expression of apoptosis-related proteins in all GI cancer cell lines. In SNU-668 cells, GE significantly reduced the anti-apoptotic protein expressions, BCL-2 and BCL-xL, while it significantly upregulated the pro-apoptotic protein Bim expression at 5 mg/mL (Fig. 4b, left panel). In Hep3B cells, GE markedly reduced the protein expressions of BCL-2 and BCL-xL at 5 mg/mL, while it upregulated Bim level without statistical significance (Fig. 4b, middle panel). In MIA PaCa-2 cells, GE significantly reduced the protein expression of BCL-2 at 5 mg/mL; however, the level of BCL-xL remained unchanged. In addition, Bim protein level was increased in a dose-dependent manner by GE treatment (Fig. 4b, right panel). In summary, GE downregulates the protein expressions involved in cell cycle regulation and anti-apoptosis, but upregulates the protein levels related to pro-apoptosis across different GI cancer cell types.

### The effects of GE on antioxidant enzyme expressions and ROS metabolism across different GI cancer cell types

Recent studies suggest that alterations in ROS metabolism influence cancer cell survival, highlighting the importance of understanding ROS regulation in cancer development and progression (23); moreover, a recent study reported that *Gochujang* significantly alters ROS metabolism in colorectal cancer cells (20).

**Fig. 5:**
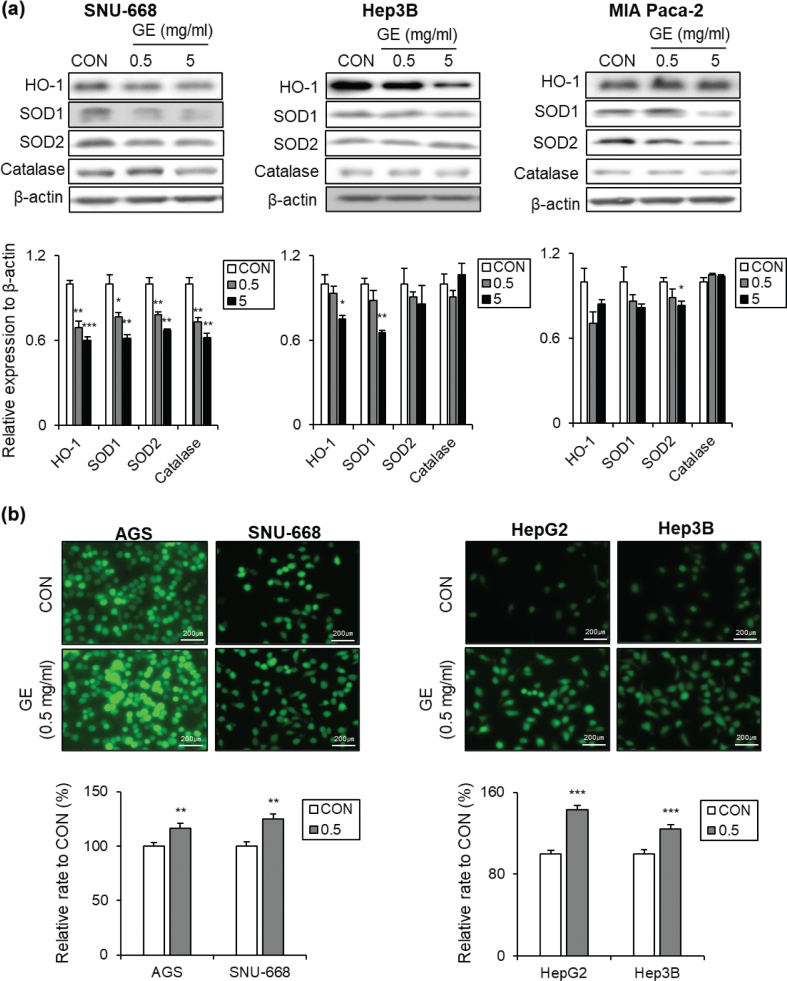
The effects of GE on the levels of antioxidant enzymes and ROS across different GI cancer cell types. (a) Antioxidant-related protein levels: Western blot images, the quantification bar graphs of SNU-668, Hep3B, and MIA PaCa-2. (b) ROS levels: Fluorescence images, the Relative bar graphs of AGS and SNU-668. *β*-Actin was used as a loading control. Values are mean ± SEM (*n* = 3). scale bar = 200 μm. A *t*-test was performed to evaluate the statistical significance of differences. * (*P* < 0.05), ** (*P* < 0.01), *** (*P* < 0.001).

As shown in Fig. 5a, GE altered the expression of antioxidant proteins across different cancer cell types. In SNU-668 cells, GE significantly reduced the protein levels of HO-1, SOD1, SOD2, and catalase in a dose-dependent manner (Fig. 5a, left panel). In Hep3B cells, GE markedly reduced HO-1 and SOD1 expression at 5 mg/mL, while SOD2 and catalase protein levels did not show significant changes, although SOD2 exhibited a slight dose-dependent decreasing trend (Fig. 5a, middle panel). In MIA PaCa-2 cells, GE showed a tendency to decrease HO-1 and SOD1 protein expressions, although the changes were not significant, while SOD2 expression was markedly reduced at 5 mg/mL. However, Catalase expression showed no difference (Fig. 5a, right panel).

Next, the effects of GE on ROS production were measured. As shown in Fig. 5b, treatment with GE for 24 h significantly increased ROS levels in gastric cancer cell lines (AGS and SNU-668) at 0.5 mg/mL (Fig. 5b, left panel). In addition, treatment with GE for 24 h at 0.5 mg/mL significantly increased ROS levels in hepatic cancer cells (HepG2 and Hep3B) (Fig. 5b, right panel). However, in pancreatic cancer cells (MIA PaCa-2), GE treatment at 0.5 mg/mL did not change ROS levels (Supplementary Fig. 3A). In summary, GE suppressed the expression of antioxidant enzymes and increased ROS production in a cell type–specific manner in different GI cancer cell types, causing an imbalance of ROS metabolism only in gastric and hepatic cancer cells.

## Discussion

The cellular and molecular mechanisms driving cancer progression can vary greatly depending on the cancer type (24, 25); therefore, the anti-cancer mechanisms of the same bioactive compound may differ across cancer cell types (8, 26). In this study, *Gochujang* significantly reduced cell viability, migration, and colony formation in various GI cancer cell lines, including gastric, hepatic, and pancreatic cancer cell lines. Moreover, *Gochujang* alters protein expression related to cell proliferation and apoptosis across all GI cancer cell types. Most interestingly, *Gochujang* significantly changes the levels of antioxidant enzymes and ROS production only in gastric and hepatic cancer cells, but not in pancreatic cancer cells. These findings present that *Gochujang* exerts anti-cancer effects through both common and cancer cell type-specific mechanisms in different GI cancer cells.

Cell viability, migration, and colony formation are widely used as indicators to evaluate the effects of anti-cancer regimens, including dietary bioactive compounds and/or foods (20, 27, 28). This study reported that *Gochujang* strongly suppressed those parameters in different GI cancer cells, confirming its significant anti-cancer effects (19). However, the reduced cell migration capability observed in this study with *Gochujang* might be attributed to decreased cell viability rather than a direct inhibition of migratory ability. Therefore, further validation using 3D spheroid assays and long-term animal studies is necessary to evaluate the actual effects of *Gochujang* on cell invasion and metastasis (29, 30). Previous studies have demonstrated that bioactive compounds exhibit cancer-cell-selective cytotoxicity by comparing their effects in non-cancerous cell lines (31, 32). Although *Gochujang* significantly reduced cell viability across multiple cancer cell lines in this study, future studies evaluating its effects in non-cancerous cell lines are strongly required to determine whether its cytotoxicity is selective for cancer cells. Furthermore, this study demonstrated *Gochujang’* s anti-cancer effects only at the *in vitro* level using relatively high treatment concentrations, limiting its physiological relevance for normal dietary intake. Therefore, future studies should validate these effects in appropriate animal models and human dietary intervention settings.

Understanding how a bioactive compound affects proliferation and apoptosis in cancer cells is critical to examining its anti-cancer effects (22). In this study, *Gochujang* markedly decreased the levels of proteins involved in cell cycle progression in various GI cancer cell lines. However, as this study compared only protein levels, the bromodeoxyuridine (BrdU) assay, which directly measures DNA synthesis and cell proliferation using flow cytometry analysis (8, 26, 33), will be helpful to confirm the findings of this study. Furthermore, *Gochujang* also strongly decreased anti-apoptotic protein levels and increased pro-apoptotic protein expressions in distinct cancer cells. Since these markers play a role in the early stage of mitochondria-mediated apoptosis, further evaluation using Annexin V/PI staining and/or analysis of apoptotic pathway factors (e.g. p53 and caspases) will be important to understand the functions of *Gochujang* in cancer cells’ apoptotic mechanisms (34, 35).

A recent study reported that *Gochujang* exerts anti-cancer effects in colorectal cancer cells by modulating ROS metabolism (20). Interestingly, this study found that *Gochujang* affects ROS metabolism differently depending on the types of GI cancer cells. *Gochujang* markedly reduced antioxidant enzyme levels in gastric and hepatic cancer cells, leading to a pronounced increase in ROS levels even at low treatment concentrations, while it did not cause any significant changes in pancreatic cancer cells. These findings suggest that *Gochujang* influences ROS metabolism through cell-type-specific mechanisms. Given the strong links between ROS metabolism and mitochondrial function (36), investigation of *Gochujang*’s effects on mitochondrial function and metabolism, including mitochondrial respiration, membrane potential, and/or its biogenesis, will be important to understand additional *Gochujang*’s anti-cancer mechanisms.

This research suggests that *Gochujang* exerts anti-cancer effects across different GI cancer cell types, including gastric, hepatic, and pancreatic cells. Similar to other anti-cancer foods, *Gochujang* commonly alters the protein expression involved in cell cycle progression and cell survival, but changes ROS metabolism cell-type dependently by regulating the levels of antioxidant enzymes and ROS production. In future studies, 3D spheroid models, additional functional assays for cell cycle regulation and apoptosis, and *in vitro* experiments are strongly required to confirm the findings in this study and finalize *Gochujang*’s potential as an anti-cancer food. Moreover, although this study focused on general antioxidant capacity, TPC, and TFC of *Gochujang*, the specific bioactive molecules driving its anti-cancer effects should be further characterized using advanced analytical techniques (e.g. HPLC or LC-MS). This compound-level profiling will be essential for elucidating the mechanisms of action of *Gochujang* and identifying candidate molecules that may underlie its selective anti-cancer activity.

## Data Availability

All of the data are available with a reasonable request from the corresponding author.
